# Hypothermia in Multiple Sclerosis: Beyond the Hypothalamus? A Case Report and Review of the Literature

**DOI:** 10.1155/2018/2768493

**Published:** 2018-03-21

**Authors:** Francesco Berti, Zeeshan Arif, Cris Constantinescu, Bruno Gran

**Affiliations:** ^1^Division of Clinical Neuroscience, University of Nottingham School of Medicine, Nottingham, UK; ^2^Department of Neurology, Nottingham University Hospitals NHS Trust, Nottingham, UK

## Abstract

Hypothermia is a rare and poorly understood complication of Multiple Sclerosis (MS). We report on a 66-year-old patient currently with Secondary Progressive MS (SP-MS) who developed unexplained hypothermia associated with multiple hospitalisations and we review the literature on this topic. In our case, magnetic resonance imaging (MRI) of the brain failed to highlight hypothalamic disease, but spinal MRI identified a number of spinal cord lesions. Given the incidence and clinical significance of spinal involvement in MS and the hypothermic disturbances observed in high Spinal Cord Injury (SCI), we hypothesise that upper spinal cord pathology, along with hypothalamic and brainstem dysfunctions, can contribute to hypothermia.

## 1. Introduction

Multiple Sclerosis (MS) is the most common demyelinating disease and the major cause of neurological disability among young adults, affecting at least 2.5 million persons worldwide [1].

The cause of MS is unknown, but strong evidence suggests it is an autoimmune disorder of the central nervous system (CNS) where a chronic inflammatory response develops against myelin autoantigens leading to demyelination, scarring, and neuronal loss [2]. The multifocal nature of the disease implies that it can occur anywhere within the white and grey matter of the brain and spinal cord; thus its symptomatology can markedly differ based on the localisation of the lesions [1].

Radiological detection of multiple plaques and areas of atrophy is suggestive of MS, but the clinical correlation is often weak, with the absence of such findings failing to explain some dysfunctions [3]. Clinical hypothermia, defined as a core body temperature below 35.0°C [4], is an example of a rare and puzzling manifestation associated with MS. Hypothalamic pathology is considered its main cause but has been radiologically identified in very few of such MS patients.

In this article, we report on an hypothermic MS patient and review the literature on this subject, focusing on exploring whether extrahypothalamic dysfunctions along the thermoregulatory network may contribute to the development of this complication.

## 2. Case Presentation

A 66-year-old lady with a 21-year history of clinically definite MS, currently in the Secondary Progressive MS (SP-MS) phase, was admitted to hospital 14 times within a two-year period. On 10 occasions this was due to unexplained symptoms such as fatigue, confusion, worsening mobility, and dysarthria associated with hypothermia and suspected urinary tract infections (UTIs) (see Table 1).

Before these admissions, the patient was clinically stable with an Expanded Disability Status Scale (EDSS) score of 6.5 and had been hospitalised only once in the previous eight years, for cellulitis in the legs. At the time, she was suffering from limb weakness (mostly in the legs), spasticity, severe fatigue, reduced hand dexterity, and blepharospasm, but no other comorbidity. She could walk for 20 metres with a supporting frame but was otherwise wheelchair dependent. Since 2005, she had been receiving Botulinum Toxin injections for lower limb spasticity and blepharospasm and was on a trial of low-dose Naltrexone. The patient was also suffering from chronic urinary retention and constipation, for which she was taking an osmotic laxative. She had established normocytic anaemia and mildly elevated liver enzymes.

After repeated admissions with hypothermia, she developed chronically low body temperature (*T*: 34.0–36.0°C) and by March 2015, she had become bed-bound for most of the day (EDSS = 8.5) and was practising intermittent self-catheterisation.

She was first found hypothermic in March 2013 after an admission for confusion (GCS 10/15), dysarthria, and reduced mobility (see Table 1). The patient had progressively deteriorated in the preceding weeks and had suffered two falls. On admission, her temperature was 34.6°C, but respiratory rate (RR), heart rate (HR), blood pressure (BP), and O_2_ saturations were unremarkable. Of note, no shivering or cold sensations were mentioned. Blood tests showed leukopenia, mild hyponatraemia (131 mmol/l) with normal K+ levels (4.6 mmol/l), and acutely elevated Liver Function Tests (LFTs) with particularly high aminotransferases. Clotting tests and spinal fluid analysis (lumbar puncture; LP) results were within normal ranges. Chest X-ray (CXR) and abdominal ultrasonography (USS) were unremarkable. Computerised tomography (CT) scan of the head showed no evidence of acute findings.

Magnetic resonance imaging (MRI) of the brain using a 3 Tesla (T) scanner was performed with and without contrast. No old brain scans were available for comparison; however bilateral, white-matter changes and generalised atrophy consistent with MS were reported. No hypothalamic involvement was detected and spinal MRI was not performed. The cause of her symptoms was not identified and the patient was treated prophylactically for Sepsis of Unknown Origin (SUO) with IV tazocin for five days. She was reviewed by general medicine and neurology and a diagnosis of Syndrome of Inappropriate Anti-Diuretic Hormone (SIADH) secretion with impaired temperature regulation secondary to MS was considered. Naltrexone was discontinued in consideration of her elevated liver enzymes. The patient gradually improved over the next three weeks and was transferred to rehabilitation services before being discharged in June with a care package.

In July 2013, she was rehospitalised because of symptoms of urinary incontinence, leg oedema, and cellulitis and was treated prophylactically for a Urinary Tract Infection (UTI) (see Table 1).

In October, she presented to the hospital with confusion, lethargy, dysarthria, worsening of movements, and decreased taste in her mouth and was found hypothermic a second time (*T*: 33.5°C) (see Table 1). Her blood results showed hyponatraemia (127 mmol/l), normokalaemia (4.4 mmol/l), decreased serum osmolality (269 mOsm/kg) with a urine osmolality of 368 mOsm/kg, and no evidence of extracellular space depletion. Her general status gradually improved while still hypothermic (*T*: 34.3°C) and after five days she was discharged. Regular monitoring of her electrolyte levels was arranged.

After the third hospitalisation with unexplained hypothermia, our patient was readmitted a fourth time in December with lethargy, dysarthria, and limb weakness (see Table 1). Once again she was found hypothermic (*T*: 33.0°C) and bradycardic. She was treated empirically for urosepsis with IV tazocin, which was then switched to nitrofurantoin as blood tests did not suggest an infectious cause. Thyroid function tests (TFTs), cortisol, Vitamin D, prolactin, parathyroid hormone (PTH), and Ca^2+^ levels were within range and a liver autoimmune screen was negative. A second brain MRI (1.5 T) showed heavy demyelinating disease burden, but no hypothalamic involvement and a likely incidental small frontal meningioma. She was discharged after two weeks, asymptomatic while still hypothermic (*T*: 34.0°C).

Between March 2014 and March 2015, our patient was hospitalised nine more times (see Table 1). Confusion, lethargy, fatigue, dysarthria, and motor weakness were the most common symptoms and associated hypothermia was registered on at least six admissions. Interestingly, on two occasions, she presented with relatively abnormal high temperatures (*T*: 37.4°C and *T*: 37.0°C).

UTI was considered the likely cause of her symptoms in six instances and antibiotics were prescribed. A three-day course of intravenous methylprednisolone was added once, with no apparent benefits and in most cases the patient recovered spontaneously. In the light of only two positive urinary samples, the absence of typical UTI symptoms, and a negative cystoscopy, urology recommended intermittent self-catheterisation due to increased residual urine volume.

**Figure 1:**
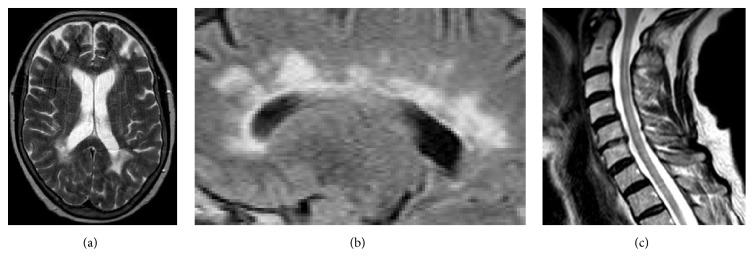
Brain and spinal MRI of the patient following admission on 12 October 2014. (a) Brain T2W axial MRI (1.5 T) demonstrating the characteristic periventricular lesions of MS. (b) Magnification of brain FLAIR sagittal MRI showing involvement of the corpus callosum. (c) Sagittal T2W spinal MRI of the cervical cord with diffuse, patchy lesions. T2W: T-2 weighted; FLAIR: Fluid-Attenuated Inversion Recovery; T: Tesla.

Following admission in October 2014, a repeat brain MRI with contrast was performed at 1.5 T that demonstrated recent callosal involvement (see Figure 1). A spinal MRI (1.5 T) was also arranged and revealed diffuse, patchy, T2 hyperintense lesions involving the majority of the cervical cord and T9-10 with associated atrophy (see Figure 1). These findings were discussed at the neuroradiology multidisciplinary team meeting and considered consistent with spinal MS rather than neuromyelitis optica spectrum disorder (NMOSD). This was confirmed by serology tests for anti-aquaporin 4 (AQP4) and anti-myelin oligodendrocyte glycoprotein (MOG) antibodies, which were both negative.

## 3. Discussion

Our patient developed clinical hypothermia (*T* < 35°C) associated with SP-MS. In the literature, this has been reported for 23 other MS cases (16 females) (see Table 2).

Most patients experienced deteriorations in cognition and consciousness (confusion, lethargy, or even stupor and coma) often accompanied by dysarthria (slurred speech) and worsening motor symptoms associated with hypothermia. On admission, their temperature ranged from 29.0°C to 35.0°C (see Table 2). In over half of these cases hypothermia had occurred after >20 years since diagnosis and was associated with severe disability (see Table 2). At least 12 of these patients suffered from more than one of such episodes (see Table 2).

Our patient developed more numerous episodes of hypothermia, superimposed on a chronic hypothermic state, than patients in previous reports. Chronic hypothermia was defined by the authors of this article as sustained hypothermia, typically lasting months. This had been previously reported in 5 other MS patients (see Table 2). In another case, chronic temperature changes were milder (35.0–36.5°C); hence this did not match the clinical definition of hypothermia [14].

Most MS patients achieved full or partial recovery after the first admission with hypothermia (see Table 2). Two deaths were associated with the initial episode [7, 12] and other two with subsequent ones [15, 17]. Transient haematological abnormalities were recorded in 16 patients during their first episode. Most commonly, these included thrombocytopenia and anaemia (see Table 2). Our anaemic patient, however, did not experience fluctuations of haematological parameters during admissions.

Previous cases of transiently deranged LFTs in hypothermic MS patients have been reported (see Table 2). In our case, mild, chronic LFT abnormalities could have been caused by Naltrexone-induced hepatic damage. Hyponatraemia was also reported in 5 of the previous cases (see Table 2). Cerebral salt wasting syndrome (CSW) and Syndrome of Inappropriate Anti-Diuretic Hormone (SIADH) both present with hyponatraemia and hyposmolality and while SIADH was suspected in this case, it was not formally confirmed. Irrespective of hypothermia, SIADH had been previously reported in MS and associated with the presence of periventricular and/or hypothalamic lesions [19, 20].

**Figure 2:**
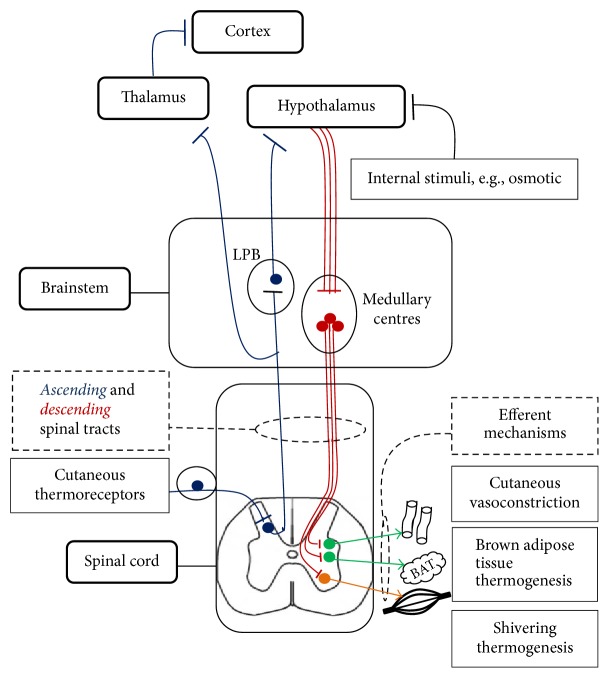
A schematic view of the main components of the thermoregulatory pathway according to the current main model [5, 6]. It is thought that cool and warm-sensitive cutaneous thermoreceptors detect changes in skin temperature. These are relayed via parallel* ascending* spinal cords tracts, to the pontine lateral parabrachial nucleus (LPB) [5]. In turn, the LPB transmits these to the anterior hypothalamus [5]. Afferent information is also separately sent to the cortex (thalamocortical tract) [5]. The hypothalamus integrates these signals with sensory information from other areas like visceral thermoreceptors and osmoreceptors to generate an effector response. In physiological conditions, after an increase in cutaneous cool signals is detected by the hypothalamic median preoptic subnucleus (MnPO) of the Preoptic Area (POA), disinhibition of the efferent pathways (in red color) leads to the activation of the three main heat-maintenance/producing mechanisms [5]. The rostral ventromedial medulla, including the rostral raphe pallidus nucleus (rRPa), is considered a key supraspinal area which regulates cutaneous vasoconstriction (CVC) and brown adipose tissue (BAT) thermogenesis (sympathetic (in green color)) and shivering thermogenesis (somatic (in orange color)) [5, 6].

More controversial is the pathophysiology of hypothermia in MS, partly because of our limited understanding of thermoregulation. Recently, however, the anatomical basis of the thermoregulatory pathways has been further characterised, mostly in rodents, which share strong similarities on thermal reflexes with humans [5]. An understanding of the current model is helpful to elucidate the importance of different areas in thermoregulation (see Figure 2).

Most of the reports on hypothermic MS patients describe deficits along the thermoregulatory circuit described (see Figure 2). For instance, our patient mentioned to be “feeling cold” only twice and, in the other 23 cases, this symptom is rarely mentioned, suggesting an impairment of the afferent tracts. Similarly, shivering and sympathetic activation (leading to CVC, BAT, and an increase in RR, HR, and BP) are considered physiological responses to mild hypothermia (32–35°C) [4] which were absent in our patient. In one of the early reports, two MS patients suffering from hypothermia were placed in a climatic chamber with a paraplegic pathological control subject [8]. They were exposed, in sequence, to environmental air temperatures of 27.0, 15.0, and 35.0°C for periods of 30–50 minutes [8]. Upon cold exposure, MS patients demonstrated cold awareness but impaired shivering and cutaneous vasoconstriction (CVC) and a small increase in the metabolic rate which resulted in a fall in core body temperature [8]. In the same conditions, the paraplegic control subject showed marked shivering and peripheral CVC, a more significant metabolic increase, and maintained core body temperature, as would be expected normally [8]. While no formal autonomic tests were arranged in our patient, no significant alterations of respiratory rate or heart rate were detected clinically or recorded in the observation charts.

Given that the hypothalamus is considered a key centre for thermoregulation, the focus of previous reports on hypothermia in MS was often on identifying hypothalamic lesions. In this case, a 3 T MRI and, subsequently, two 1.5 T MRI scans with contrast failed to detect hypothalamic involvement. Brain MRI was recorded in 15 other hypothermic MS patients, but radiological evidence for hypothalamic involvement was poor (*n* = 2; 13%) and in both cases it involved the preoptic area (POA) [17, 18]. Out of three brains which were examined postmortem, hypothalamic pathology was evident in two [9, 12, 14] (see Table 2). Previous to autopsy, brain MRI had been performed in one of such cases but had failed to detect hypothalamic changes, despite identifying periventricular lesions [14]. Independently, an MRI study on 105 Caucasian patients, with clinically definite MS without hypothermia and typical lesions, revealed a similar (13%) frequency of radiologically-detectable hypothalamic changes, using a 1.5 Tesla MRI scanner, with conventional protocols [21]. Instead, a postmortem study on 17 nonhypothermic MS patients found hypothalamic lesions in 16 brains (97%), 60% of which showed active inflammation [22]. Different factors may explain this disparity in results. Firstly, poor radiological sensitivity, particularly in the earlier reports on hypothermia in MS, may account for the low presence of hypothalamic lesions. Secondly, the patient cohorts of Qiu et al. [21] and Huitinga et al. [22] were different, with the latter having a greater mean age of disease duration which was statistically associated with a greater number of active hypothalamic lesions [22]. Although, using the current MR technology, we are unable to exclude very small hypothalamic lesions, we are mindful that the latter have not been found in other reported cases [14] and by contrast, they can be present in MS patients not affected by hypothermia.

Together with hypothalamic changes, callosal, brainstem, and spinal cord lesions were also detected at autopsy in hypothermic MS patients (see Table 2). All these areas have been previously associated with the development of hypothermic episodes and both the brainstem and the upper spinal cord are known as important thermoregulatory centres [4, 5, 14] (see Figure 2).

Brain callosal involvement, for instance, was detected in our case (see Figure 1) and in other four hypothermic MS patients via MRI or autopsy (see Table 2). In one instance, this was associated with hypothalamic disease [18]. In another report, MRI hyperintensities in the right posterior thalamus were associated with generalised atrophy, displaying clinical similarities to Shapiro's syndrome [16]. This is characterised by the congenital agenesis of the corpus callosum, hyperhidrosis, and recurrent hypothermia [23].

Brainstem lesions were associated with hypothermia in two other MS cases [4, 14]. In addition, a mesodiencephalic haematoma has been reported to be associated with hypothermia in a non-MS patient [24].

Upper spinal cord pathology in MS could also be associated with hypothermia. Spinal involvement of the cervical region is particularly common in MS and involves both white and grey matter, interneurons and motoneurons [25, 26]. Similarly to brainstem lesions, upper spinal cord changes could impair both ascending and descending tracts of the thermoregulatory circuit (see Figure 2). Extensive spinal cord lesions associated with brain and hypothalamic involvement were found at autopsy in one MS patient [12] and were radiologically detected in ours (see Figure 1). In the previously reported cases, however, spinal MRI was never reported and in ours it was only performed once, after repeated episodes of hypothermia. Hence, our ability to directly estimate the impact of spinal lesions on the development of hypothermia in MS is limited.

Given the prominent spinal involvement, the differential diagnosis of neuromyelitis optica spectrum disorder (NMOSD) was discussed at a neuroradiology meeting but was ruled out on the basis of clinical presentation, radiological features (multiple, confluent patchy lesions rather than longitudinally extensive lesions), and serology results (anti-AQP4 and anti-MOG antibody negative) [27].

Of note, hypothermia with autonomic impairment is commonly observed after upper Spinal Cord Injury (SCI) [28, 29]. A retrospective study of 50 tetraplegic patients found that subnormal core body temperatures (35.0–36.4°C) were present in all patients and clinical hypothermia was recorded in 15 [30]. Similarities between dysfunctional sympathetic sudomotor skin responses were also identified among patients with transection of the SC at different levels and MS patients [31]. In spite of these resemblances and the frequent involvement of the spinal cord in MS, spinal lesions have not been reported to cause hypothermia in MS. This may be because, similarly to other lesions, a critical impairment of conduction is required before symptoms become manifest and in MS, unlike after SCI, this process is progressive and difficult to monitor.

Interestingly, our patient developed two episodes of abnormally high temperatures (above 36.5°C), associated with admissions (see Table 1). A clinical decay at high temperatures has been previously documented in MS patients (“Uhthoff's phenomenon”) but, to our knowledge, was never reported to cause admissions in hypothermic individuals. This effect likely stems from decreased axonal conduction in damaged nerves at higher temperatures [32, 33]. Why this phenomenon occurs at lower temperatures in chronically hypothermic MS patients is controversial. This may indicate a more severe axonal damage or simply the resetting of the body thermostat at a new lower point where these higher temperatures are considered extreme [4, 17].

Regardless of the causative mechanisms, no effective strategies have been devised to treat and prevent the development of hypothermic episodes in MS patients. Antibiotic treatment, in the absence of signs of infection, did not show any objective benefit for our patient and is known to promote antimicrobial resistance. Spontaneous recovery was commonly reported [18]. Treatment with steroids was shown to be potentially beneficial [17] but in our experience did not lead to substantial improvements. Our patient used an electrical blanket to control her body temperature at home, but the usefulness of this measure has not been systematically assessed. However, its use seems logical to prevent hypothermia since the neurological impairments along the thermoregulatory circuit.

## 4. Conclusion

In summary, hypothermia in MS patients remains a poorly understood phenomenon. The anatomical location of the causative lesions remains controversial and, based on the available evidence [4–6], we hypothesise that upper spinal cord, as well as brain stem lesions, may be involved in its pathogenesis in MS, independently of hypothalamic pathology. Given the disseminated nature of the disease, multiple, anatomically distinguished lesions, as opposed to a large single lesion, may also contribute to the development of this advanced complication by disrupting the thermoregulatory network at different levels [18]. In our opinion, in hypothermic MS patients, spinal MRI should be added to brain MRI to verify the presence of spinal involvement, due to its clinical importance. With the development of more sensitive neuroimaging and follow-up scans, anticipating the clinical course of hypothermia in these patients may be possible. Currently, in fact, the development of chronic hypothermia remains unpredictable.

## Figures and Tables

**Table 1:** Summary of patient admissions to hospital between March 2013 and March 2015.

Admission date	Main complaint(s)	Temperature at admission (°C)	New finding(s)	Confirmed diagnosis	Treatment(s)	Disease course	Neuroimaging
24 March 2013	Confusion, dysarthria, reduced mobility, and recent falls	34.6	GCS (10/15) Leukopaenia Hyponatraemia (Na+ 131 mmol/l), mildly deranged LFTs Normal LP, CXR, and abdominal USS	No ?SUO ?SIADH	IV antibiotics, naltrexone discontinued because of LFTs	Discharged in 3 weeks (homeothermic) with care package and rehabilitation	Head CT and brain MRI. No acute findings. Bilateral, white-matter changes and generalised atrophy. No hypothalamic involvement

18 July 2013	Urinary incontinence, oedema, and cellulitis	35.8	No	No ?UTI	Antibiotics	Discharged	No

18 October 2013	Confusion lethargy, dysarthria, worsening movements, and decreased taste	33.5	Nystagmus Diplopia Decreased limb power Hyponatraemia (127 mmol/l), normokalaemia (4.4 mmol/l) Hyposmolarity (serum osmolality: 269 mOsm/kg; Urine Osmolality: 368 mOsm/kg)	No ?SIADH	Supportive	Discharged in 5 days while still hypothermic (*T*: 34.3°C)	No

7 November 2013	Dizziness on standing	33.0	No	No	Supportive	Discharged	No

31 December 2013	Lethargy, unwell, dysarthria, and limb weakness	33.0	Bradycardia, normal liver autoimmune screen, Vitamin D, TFTs, prolactin, PTH, calcium, and random cortisol	No ?UTI	Antibiotics	Discharged in 2 weeks (*T*: 34.0°C)	Brain MRI: no acute findings. Heavy demyelinating disease burden and a likely incidental small frontal meningioma. No hypothalamic involvement

16 March 2014	Feeling cold, unwell, and dysarthria	32.8	RUQ tenderness Murphy's +ve. Abdominal USS: cholelithiasis and contracted gall bladder	No	Antibiotics then supportive	Discharged in 2 weeks	No

22 May 2014	Weakness	33.1	Positive MSU, CRP 41	UTI	Antibiotics	Discharged the next day	No

27 May 2014	Feeling cold and weakness	33.7	No	No	Supportive	Discharged in 3 days	No

15 September 2014	Right flank pain	32.7	Urinalysis (positive for leukocites and blood +++)	UTI	Antibiotics	Discharged in a week	No

2 October 2014	Right flank pain, urinary incontinence, confusion, and persistently low temperatures	34.0	No	AKI and ?UTI	Antibiotics	Discharged in 6 days	No

12 October 2014	Dysarthria, fatigue, confusion, weakness, and decreased power	NK	Hyponatraemia, hyperkalaemia (Na+ 125 mmol/l, K+ 5.8 mmol/l), eGFR 59, urea 8.4 mmol/l MSU (positive for leukocytes) Mixed growth, possible contamination	UTI	Antibiotics 3 days of IV steroids	Discharged in 2 weeks	Brain and spinal MRI Extensive demyelinating lesions with evidence of recent callosal involvement. Spinal imaging revealed diffuse, patchy, T2 hyperintense lesions involving the majority of the cervical cord and T9-10 with associated atrophy

24 October 2014	Neck pain, fatigue, and weakness	37.4	No	No ?UTI	Antibiotics	NK	No

23 March 2015	Lethargy	31.0	No	No? UTI	Supportive	Discharged on same day	No

25 March 2015	Lethargy and high-temperature	37.0	Dysmetric saccades. Cerebellar signs. Worsening power with bilateral upgoing plantar reflexes. Bilateral lower leg oedema.	No	Supportive	Discharged 2 days later	No

Not known (NK); Glasgow Coma Scale (GCS); acute kidney injury (AKI); mid-stream urine (MSU); C-reactive protein (CRP); right upper quadrant (RUQ); sepsis of unknown origin (SUO); liver function tests (LFTs); lumbar puncture (LP); chest X-ray (CXR); ultrasonography (USS); thyroid function tests (TFTs); parathyroid hormone (PTH); estimated glomerular filtration rate (eGFR); Syndrome of Inappropriate Anti-Diuretic Hormone (SIADH).

**Table 2:** Review of the literature: summary of the 1st presentation of patients with hypothermia in Multiple Sclerosis (MS) and the clinical course of the disease.

References	Patient details	Disease duration; MS-type; EDSS	Main complaint(s) before admission	Temperature at admission (°C)	Cognitive symptoms at admission	New neurological signs and symptoms	Dysarthria and/or dysphagia	Haematological abnormalities and onset	Hyponatremia and plasma/urinary osmolalities	Neuroimaging and/or autopsy studies	Suspected diagnosis and disease course	Type of hypothermia and (number of hypothermic episodes)
[7]	61 F	NK; NK; NK	Lethargy, anorexia, poor fluid intake	29.4	NK	NK	NK	Hb 12.9 g/dl; MCV 84 fl Platelets 19 × 109/l Bone marrow aspirate: erythroid hypoplasia	NK	No	Death	Acute (1)

[8]	41 F	7 years; NK; EDSS: 7.0	3 weeks confusion, apathy	32.6	Confusion, Stupor	Marked rigidity in all limbs	No	After 1/52: Anaemia (Hb 7.9 g/dl) Thrombocytopenia (61 × 10^9^/l)	No	Head CT: no abnormality detected	Treated with passive rewarming. Full clinical recovery in 6 days	Chronic: (1)

[8]	52 F	24 years; NK; EDSS: 8.0	3 weeks: confusion, lethargy	31.0	Coma	No	No	Thrombocytopenia (50 × 10^9^/l) at admission, peaking after 5 days (28 × 10^9^/l) and anaemia (Hb 7.4 g/dl)	Yes: (Na+ 107 mmol/l) (?SIADH)	No	Treated with steroids, passive rewarming, hypertonic saline, and furosemide. Full clinical recovery in 5 days	Chronic: (1)

[9]	55 F	24 years; NK; EDSS: 6.0	1 week: confusion, lethargy, visual hallucinations	33.0	Confusion	Generalised myoclonus neck stiffness	No	8 days after: pancytopenia Hb 9.2 g/dl, Platelet 80 × 10^9^/l, Leukocytes 2.9 × 10^9^/l	No	Head CT: no abnormality detected Brain autopsy: multiple old plaques at various locations (incl. basal ganglia, corpus callosum, occipital white matter, and right upper cerebellar peduncle). No hypothalamic lesions except some recent axon swellings and cell loss	Developed bronchopneumonia. Treated with antibiotics. Full clinical recovery	Acute: (4)

[9]	55 F	22 years; NK; NK	4 weeks: confusion, bradyphrenia, incontinence	NK	NK	Augmented paraparesis	No	No	No	Brain MRI and CT performed after the 2nd admission with hypothermia. Brain MRI and head CT: Important lesions in periventricular and posterior part of corpus callosum No hypothalamic lesions	Developed bronchopneumonia. Treated with antibiotic and passive rewarming. Full clinical recovery.	Acute: (2)

[9]	58 M	16 years; NK; EDSS: 7.0	2 weeks: lethargy, dysarthria, dysphagia	<35	Memory deficit	Tetraparesis, bilateral central nystagmus	Dysarthria, dysphagia	Thrombocytopenia: 100 × 10^9^/l	No	Brain MRI performed after the 4th admission with hypothermia. Brain MRI: several periventricular plaques. No hypothalamic lesions	Developed bronchopneumonia. Treated with antibiotics and passive rewarming recovered in days. Some motor deterioration remained	Acute: (4)

[10]	52 M	14 years; NK; NK	Augmented motor deficits	32.8	Confusion	Augmented motor paresis	No	No	Yes: (Na+ 114 mEq/l), plasma hyposmolarity (? SIADH)	Brain MRI: No hypothalamic lesions	Treatment with NaCl infusion and fluid restrictions. Hypothermia self-resolved. Clinical full recovery	Acute: (3)

[11]	63 F	25 years; NK; NK	Visual disturbances, depression paranoid, unable to stand up without help	32.4	Confusion	Worsening neurological signs: bilateral Babinski sign, paraparesis, paresthesia, and ataxia in the right arm and mild postural tremor	Dysarthria	Deranged LFTs (ALT and AST mildly raised with hypoalbuminaemia, 31.7 g/l)	No	Brain MRI: diffuse white matter lesions No hypothalamic lesions	Treated with passive rewarming and parenteral thiamine (? Wernicke Encephalopathy) Normothermia in 3 weeks	Acute (1)

[11]	68 F	32 years; NK; NK	3 weeks: gait abnormalities, dysarthria	31.6	Confusion, drowsiness	Severe paraparesis, bilateral Babinski sign, asterixis, partial right lateral rectus palsy, cerebellar signs	Dysarthria	Severe hypoalbuminaemia (18.9 g/l), decreased folic acid	No	Brain MRI: multiple periventricular lesions. No hypothalamic lesions	Treated with parenteral thiamine (? Wernicke Encephalopathy). Full recovery within 1 month	Acute (1)

[12]	53 F	NK; NK; NK	5 days: lethargy, dysphagia, dysarthria	29.0	Confusion	Spastic tetraparesis with bilateral extensor plantar but depressed reflexes	Dysarthria	Thrombocytopenia: platelets 79 × 10^9^/l Increased APTT ?DIC Raised amylase (321 IU/l)	No.	Brain and spine autopsy: multiple plaques in the brain and spinal cord. A large hypothalamic plaque was found with evidence of current activity and demyelination	Passive rewarming, antibiotics, and atropine. Developed bronchopneumonia, pancreatitis, and died	Acute (1)

[13]	44 F	10 years; PR-MS; NK	Few days: confusion, disorientation, hallucinations	33.3	Confusion	Flaccid paraplegia and cerebellar syndrome (not augmented)	No	No	No	Brain MRI: T2W hyperintensities in the periventricular white matter	Passive rewarming and full clinical recovery within 10 days	NK

[14]	48 M	5 years; NK; EDSS: 6.0	3 weeks: confusion, disorientation, dysarthria deteriorating mobility, drowsiness, cold lower extremities	Initially 36.0 then 31.0	Stupor	Initially flaccid paraparesis and increased tone in the upper limbs. Deterioration over 48 h. He developed repetitive facial twitching, neck stiffness, left lower motor facial weakness, and decerebrate posturing	Dysarthria	Thrombocytopenia: 27 × 10^9^/l Anaemia: Hb 12.7 g/dl. Increased PT and APTT and low folate	No	CT head: moderate brain atrophy. Previous MRIs had been normal. Brain MRI: after 1st and 2nd admissions with hypothermia: multiple high signals in periventricular white matter. No hypothalamic lesions. Brain autopsy: plaques in periventricular, midbrain, pons, medulla and hypothalamus (incl. posterior hypothalamic nucleus)	Initially treated with IV methylprednisolone for MS relapse, then with antibiotics for ?UTI. Then, passive rewarming. Normothermia after further 48 h. Packed cells, platelets, and plasma proteins transfusion for bleeding. Discharged in 30 days. Residual spastic paraparesis, incoordination, mild upper limb weakness, and sensory deficit after T12	Acute then chronic: (2)

[14]	59 M	30 years; NK; EDSS: 7.0	4 weeks: increasing fatigue, lethargy, confusion, then drowsiness, dysphagia, and dysarthria	33.0	Stupor	NK	Dysphagia, dysarthria	Thrombocytopenia 95 × 10^9^/l	No	NK	Passive rewarming and IV fluids. Normothermia in 36 hours. Paranoid psychosis and confusion, MI and severe LVF. Residual cognitive impairment	Acute (2)

[14]	57 F	20 years; NK; EDSS: 7.0	Decreased mobility, lethargy, dysphagia,	35.0	Oriented (initially)	Bilateral optic atrophy and absent oculocephalic response, neck stiffness, rigidity, spastic tetraparesis	Dysarthria, dysphagia	Thrombocytopenia. when normothermic (141) then 99 × 109/l. Raised APTT time. Raised platelets antibodies	Yes: (Na+ 130 mmol/l)	Head CT: bilateral periventricular low density lesions	Rewarming, IV fluids and IV methylprednisolone and antibiotics for ?UTI and respiratory infections were given. Normothermia within 24 hours. Full clinical recovery in 4 weeks	Acute: (2)

[14]	64 F	30 years; NK; EDSS: 9.0	Deterioration of motor function, speech disturbance, peripheral oedema, fluctuating consciousness	34.7	Confusion	Periorbital oedema, augmented tetraparesis, impaired palatal movements	Dysarthria	No	Yes: (Na+ 130 mmol/l) corrected with fluid restriction. Normal plasma and urinary osmolalities	NK	Passive rewarming. Normothermia in 24 hours. Full clinical recovery in 7 days	Acute (1)

[14]	47 F	No previous MS (diagnosed in retrospective); EDSS: 3.0	Withdrawal and lethargy	29.0	Coma	Neck stiffness, generalised hypertonia. After 3 days: bilateral extensor plantar responses, mild paraparesis, optic disc pallor	NK	Thrombocytopenia: 33 × 10^9^/l Anemia: Hb 10.2	No	Brain MRI: diffuse cortical atrophy, T2W hyperintense periventricular lesions. No hypothalamic lesions	Rewarming. Normothermia in 3 days. Residual physical and cognitive deficits	Acute: (2)

[15]	NK F	NK; NK; NK	Motor and cognitive decline	NK	Drowsiness	Augmented flaccid paresis	Dysarthria	Thrombocytopenia	NK	Head CT and brain MRI: No hypothalamic lesions	NK	Chronic with acute episode: (2)

[15]	NK F	NK; NK; NK	Motor and cognitive decline	NK	Drowsiness	Augmented flaccid paresis	Dysarthria	Thrombocytopenia	NK	Head CT and brain MRI: No hypothalamic lesions.	NK	Chronic with acute episodes (NK)

[16]	45 F	28 years; SP- MS; EDSS: 8.0	4 weeks: hypothermia (32-33°C), stupor, hypotension, hyponatraemia, and hypoglycaemia	33.4	Stupor	No	Dysarthria	Chronic normocytic anaemia. Elevated APTT (61 s). Raised CRP with negative blood cultures. Hypoglycaemia	Yes: (124 mmol/l). ?CSW syndrome	Head CT and brain MRI: known right parietal defect (previous brain abscess), generalised atrophy, periventricular white matter lesions. particularly in the callosum and a hyperintense lesion in the septal region of right thalamus. No hypothalamic lesions	Antibiotics, IV fluids. Initially recovered then further deterioration within a week (33.1°C) stupor and severe hypotension. Within 2 weeks a 3rd episode of hypothermia (31.2°C), bradycardia, and hypotension. She was treated with droxidopa and then discharged once normothermic and stable	Acute: (6)

[4]	61 F	30 years; SP-MS; NK	Confusion, agitation	33.9	Confusion, agitation	No	No	No	No	Brain MRI performed after 3rd hypothermic episode. Periventricular and brain stem plaques were seen with small vessel ischaemia in the ganglionic regions. No hypothalamic involvement	Spontaneous improvement and discharge with a T of 35.2°C	Chronic with acute episodes (6)

[17]	41 M	7 years; NK; NK	3 weeks: slurred speech, hypothermia, dysarthria, paranoid delusions, auditory, visual and tactile hallucinations	30.0	Confusion then coma	Bilateral facial droop, miosis, paraplegia (also present before), and bilateral upper extremities weakness	Dysarthria	Platelets: 113000/mm^3^	No	Brain MRI: increased overall lesions and new T2W hypothalamic hyperintensity	Passive rewarming. Then antibiotics and respiratory assistance for ?SUO. Full clinical recovery in 6 weeks. Five monthly IV methylprednisolone infusions (1 g/month)	Acute (6)

[18]	39 M	24 years; SP-MS; EDSS: 6.5	Few weeks: augmented spasticity, cognitive decline, confusion	31.0	Stupor	Spastic tetraparesis	Dysarthria, dysphagia	Thrombocytopenia (75 × 10^9^/l) Leukopenia (0.7 × 10^9^/l) Elevated APTT (37 s) Raised ALT and AST	No	Brain MRI: T2W multiple white matter lesions and atrophy of corpus callosum. Hypothalamic involvement with bilateral nonenhancing preoptic lesions. After 1 year MRI showed no longer signs	Full clinical recovery	NK

[18]	49 M	32 years; PP-MS; EDSS: 7.5	Confusion	32.4	Psychomotor slowing	Augmented tetraparesis, bilateral pyramidal syndrome, right cerebellar syndrome	Dysarthria, dysphagia	Thrombocytopenia (79 × 10^9^/l) Raised AST and ALT	No	Brain MRI: T2W white matter lesions. No hypothalamic involvement	Antibiotics for sepsis and 3 steroid injections. Full clinical recovery within days	Acute (1)

Expanded Disability Status Scale (EDSS); Primary Progressive MS (PP-MS); Secondary Progressive MS (SP-MS); not known (NK); aspartate transaminase (AST); alanine transaminase (ALT); disseminated intravascular coagulation (DIC); activated partial thromboplastin time (APTT); Syndrome of Inappropriate Anti-Diuretic Hormone (SIADH); mean corpuscular volume (MCV); T-2 weighted (T2W).

## References

[1] Dendrou C. A., Fugger L., Friese M. A. (2015). Immunopathology of multiple sclerosis. *Nature Reviews Immunology*.

[2] Goldenberg M. M. Multiple Sclerosis Review. http://www.pubmedcentral.nih.gov/articlerender.fcgi?artid=3351877&tool=pmcentrez&rendertype=abstract.

[3] Vellinga M. M., Geurts J. J. G., Rostrup E. (2009). Clinical correlations of brain lesion distribution in multiple sclerosis. *Journal of Magnetic Resonance Imaging*.

[4] Alty J. E., Ford H. L. (2008). Multi-system complications of hypothermia: A case of recurrent episodic hypothermia with a review of the pathophysiology of hypothermia. *Postgraduate Medical Journal*.

[5] Morrison S. F. (2016). Central neural control of thermoregulation and brown adipose tissue. *Autonomic Neuroscience: Basic & Clinical*.

[6] Nakamura K. (2011). Central circuitries for body temperature regulation and fever. *American Journal of Physiology-Regulatory, Integrative and Comparative Physiology*.

[7] O'Brien H., Amess J. A., Mollin D. L. (1982). Recurrent thrombocytopenia, erythroid hypoplasia and sideroblastic anaemia associated with hypothermia. *British Journal of Haematology*.

[8] Sullivan F., Hutchinson M., Bahandeka S., Moore R. E. (1987). Chronic hypothermia in multiple sclerosis. *Journal of Neurology, Neurosurgery & Psychiatry*.

[9] Lammens M., Lissoir F., Carton H. (1989). Hypothermia in three patients with multiple sclerosis. *Clinical Neurology and Neurosurgery*.

[10] Ghawche F., Destée A. (1990). Hypothermia and multiple sclerosis. A case with 3 episodes of transient hypothermia. *Nature Reviews Neurology*.

[11] Geny C., Pradat P. F., Yulis J., Walter S., Cesaro D., Degos J. D. (1992). Hypothermia, Wernicke encephalopathy and multiple sclerosis. *Acta Neurologica Scandinavica*.

[12] Edwards S., Lennox G., Robson K., Whiteley A. (1996). Hypothermia due to hypothalamic involvement in multiple sclerosis. *Journal of Neurology, Neurosurgery & Psychiatry*.

[13] Mouton P., Woimant F., Ille O. (1996). Hypothermia and the nervous system. Review of the literature apropos of 4 cases. *Annales De Medecine Interne*.

[14] White K. D., Scoones D. J., Newman P. K. (1996). Hypothermia in multiple sclerosis. *Journal of Neurology, Neurosurgery & Psychiatry*.

[15] Feneberg W., König N. H. (2006). Two cases of hypothermia in multiple sclerosis. *Journal of Neurology*.

[16] Linker R. A., Mohr A., Cepek L., Gold R., Prange H. (2006). Core hypothermia in multiple sclerosis: Case report with magnetic resonance imaging localization of a thalamic lesion. *Multiple Sclerosis Journal*.

[17] Weiss N., Hasboun D., Demeret S. (2009). Paroxysmal hypothermia as a clinical feature of multiple sclerosis. *Neurology*.

[18] Darlix A., Mathey G., Monin M-L. (2012). Hypothalamic involvement in multiple sclerosis. *Nature Reviews Neurology*.

[19] Ishikawa E., Ohgo S., Nakatsuru K. (1989). Syndrome of Inappropriate Secretion of Antidiuretic Hormone (SIADH) in a Patient with Multiple Sclerosis. *Japanese Journal of Medicine *.

[20] Liamis G., Elisaf M. (2000). Syndrome of inappropriate antidiuresis associated with multiple sclerosis. *Journal of the Neurological Sciences*.

[21] Qiu W., Raven S., Wu J. (2011). Hypothalamic lesions in multiple sclerosis. *Journal of Neurology, Neurosurgery & Psychiatry*.

[22] Huitinga I., De Groot C. J., Van der Valk P., Kamphorst W., Tilders F. J., Swaab D. F. (2001). Hypothalamic lesions in multiple sclerosis. *Journal of Neuropathology & Experimental Neurology*.

[23] Shapiro W. R., Williams G. H., Plum F. (1969). Spontaneous recurrent hypothermia accompanying agenesis of the corpus callosum. *Brain*.

[24] Gaymard G., Cambon H., Dormont D., Richard A., Derouesne C. (1990). Hypothermia in a mesodiencephalic haematoma. *Journal of Neurology, Neurosurgery & Psychiatry*.

[25] Gilmore C. P., Geurts J. J. G., Evangelou N. (2009). Spinal cord grey matter lesions in multiple sclerosis detected by post-mortem high field MR imaging. *Multiple Sclerosis Journal*.

[26] Lukas C., Sombekke M. H., Bellenberg B. (2013). Relevance of spinal cord abnormalities to clinical disability in multiple sclerosis: MR imaging findings in a large cohort of patients. *Radiology*.

[27] Wingerchuk D. M., Banwell B., Bennett J. L. (2015). International consensus diagnostic criteria for neuromyelitis optica spectrum disorders. *Neurology*.

[28] Menard M., Hahn G. (1991). Acute and chronic hypothermia in a man with spinal cord injury: environmental and pharmacologic causes. *Archives of Physical Medicine and Rehabilitation*.

[29] Colachis S. C. (2002). Hypothermia associated with autonomic dysreflexia after traumatic spinal cord injury. *American Journal of Physical Medicine & Rehabilitation*.

[30] Khan S., Plummer M., Martinez-Arizala A., Banovac K. (2007). Hypothermia in patients with chronic spinal cord injury. *The Journal of Spinal Cord Medicine*.

[31] Yokota T., Matsunaga T., Okiyama R. (1991). Sympathetic skin response in patients with multiple sclerosis compared with patients with spinal cord transection and normal controls. *Brain*.

[32] Uhthoff W. (1890). Untersuchungen über die bei der multiplen Herdsklerose vorkonimenden Augenstörungen. *Archiv für Psychiatrie und Nervenkrankheiten*.

[33] Davis S. L., Frohman T. C., Crandall C. G. (2008). Modeling Uhthoff's phenomenon in MS patients with internuclear ophthalmoparesis. *Neurology*.

